# Completeness and regularity of generalized fuzzy graphs

**DOI:** 10.1186/s40064-016-3558-6

**Published:** 2016-11-15

**Authors:** Sovan Samanta, Biswajit Sarkar, Dongmin Shin, Madhumangal Pal

**Affiliations:** 1 Department of Industrial and Management Engineering, Hanyang University, Ansan, Gyeonggi 15588 South Korea; 2 Department of Applied Mathematics with Oceanology and Computer Programming, Vidyasagar University, Midnapore, 721 102 India

**Keywords:** Generalized fuzzy graphs, Effective edge, Regular graphs, Complete graphs, Matrix

## Abstract

Fuzzy graphs are the backbone of many real systems like networks, image, scheduling, etc. But, due to some restriction on edges, fuzzy graphs are limited to represent for some systems. Generalized fuzzy graphs are appropriate to avoid such restrictions. In this study generalized fuzzy graphs are introduced. In this study, matrix representation of generalized fuzzy graphs is described. Completeness and regularity are two important parameters of graph theory. Here, regular and complete generalized fuzzy graphs are introduced. Some properties of them are discussed. After that, effective regular graphs are exemplified.

## Background

Nowadays, graphs do not represent all the systems like networks, routes, schedules, images, etc. properly due to the uncertainty or haziness of the parameters of systems. For example, a social network may be represented as a graph, where vertices represent an account (person, institution, etc.) and edges represent the relation between those accounts. If the relations among accounts are measured as either good or bad according to the frequency of contacts among those accounts, then fuzzyness can be added for such representations. This and many other problems lead to define fuzzy graphs. The first definition of a fuzzy graph was introduced by Kauffman (1973). But, Rosenfeld (1975) described fuzzy relations on fuzzy sets and developed some theory of fuzzy graphs. Using these concept of fuzzy graphs, Koczy (1992) discussed fuzzy graphs to evaluate and to optimize any networks. Samanta and Pal (2013) showed that fuzzy graphs can be used in competition in ecosystems. After that, they introduced some different types of fuzzy graphs (Samanta and Pal 2015; Samanta et al. 2014). Bhutani and Battou (2003) and Bhutani and Rosenfeld (2003) discussed different arcs in fuzzy graphs. For further details of fuzzy graphs, readers may look in Mathew (2009), Mordeson and Nair (2000), Pramanik et al. (2014, 2016) and Rashmanlou et al. (2015). Applications of fuzzy graph include data mining, image segmentation, clustering, image capturing, networking, communication, planning, scheduling, etc.

Sunitha (2001) introduced and discussed some properties of complete fuzzy graphs. The same technique as in crisp complete graph, is used. After that, Parvathi and Karunambigai (2006) extended the concept of fuzzy graphs to introduce intuitionistic fuzzy graphs. Here, membership values and non-membership values are associated. Nagorgani and Radha (2012) defined regular fuzzy graphs in which the degree of vertices are assumed as same as in crisp graphs. Akram (2011) introduced interval valued fuzzy graphs. In that paper, vertex and edge membership values are taken as intervals but the same technique applied for edge restriction as in fuzzy graphs. After that, Akram and Dudek (2011) introduced bipolar fuzzy graphs. Here, positive and negative membership values of vertices and edges are taken. Kittur (2012) and Radha and Kumaravel (2014) described some important properties of complete and regular fuzzy graphs.

In all these fuzzy graphs, there is a common property that edge membership value is less than to the minimum of it’s end vertex membership values. Suppose, a social network is to be represented as fuzzy graphs. Here, all social units are taken as fuzzy nodes. The membership values of the vertices may depend on several parameters. Suppose, the membership values are measured according to the sources of knowledge and the relation between those units is represented by fuzzy edges. Thus, the membership value is measured according to the transfer of knowledge. But, transfer of knowledge may be greater than one of the social actors/units as more knowledgeable person informs less knowledgeable person. But, this concept cannot be represented in fuzzy graphs as edge membership value should be less than membership values of end vertices. Thus, all images/networks cannot be represented by fuzzy graphs. To remove the restriction, generalized fuzzy graphs are introduced here.

Matrix representation is another way of representations. Different authors established different properties of fuzzy graph matrices. Recently, Khansamy and Thangaraj (2015) discussed about some properties of matrices of fuzzy graphs. In this research, generalized fuzzy graphs are represented by appropriate matrices. Sunitha (2001) discussed different major properties of fuzzy graphs in her research work. She described fuzzy complete graphs, regular graphs and many more. Kittur (2012) provided some properties on complete fuzzy graphs. Nagorgani and Radha (2012), Nagorgani and Latha (2008) and Radha and Kumaravel (2014) described some of the properties of regularity and irregularity of fuzzy graphs. These studies are regular extension of crisp graphs. In this paper, regularity is defined in more generalized way.

After introductory section, generalized fuzzy graphs of type 1 and type 2 (GFG1, GFG2) are described with suitable examples. After that, GFG1 and GFG2 are represented by matrices. Complete GFG1, GFG2 are introduced. Then, regular and effective regular GFG1, GFG2 are introduced and several properties are established. At last, conclusions are given.

## Problem definition of this work

The direction of this work is to generalize the fuzzy graphs by removing the edge restriction. The relation between vertices and edges are to be established. Two properties, completeness and regularity, are to be discussed.

## Generalized fuzzy graphs

A fuzzy graph $\xi =(V,\sigma ,\mu )$ is a non-void set *V* with a pair of functions $\sigma :V\rightarrow [0, 1]$ and $\mu :V \times V \rightarrow [0,1]$ such that for each $x, y\in V$, $\mu (x,y) \le \sigma (x)\wedge \sigma (y)$, where $\sigma (x)$ and $\mu (x,y)$ represent the membership values of the vertex *x* and edge (*x*, *y*) in $\xi$ respectively.

Now, generalized fuzzy graphs are to be defined. The membership value of vertices of graphs depend on membership values of the adjacent edges. The membership values of isolated vertices are taken as 0. The membership function is defined from a non-void set to a closed interval [0, 1]. Thus, any linguistic term can be defined by membership values. Some times, vertex membership values are considered first and depending on vertex membership values, the edge membership values are assumed. For example, social networks, where social actors and its stability are considered first. Depending on stability, vertex membership values are determined. After that, relation among the actors are considered. The membership values may be taken from the parameter ‘relationship’. In some real problems, edges are considered first and depending on edge membership values the vertex membership values are considered. For example, capacities of pipelines can be taken as edge membership values and depending on the capacities, vertex membership values are decided (Table 1).

**Table 1:** Authors contributions towards generalized fuzzy graphs

Authors	Year	Contributions
Kauffman (1973)	1973	Introduction of fuzzy graphs
Rosenfeld (1975)	1975	Modification of the concept of fuzzy graphs given by Kauffman (1973). He added that edge membership value is less than minimum of vertex membership values
Sunitha (2001)	2001	Introduction and discussion of properties of complete fuzzy graphs
Parvathi and Karunambigai (2006)	2006	Introduction of intuitionistic fuzzy graphs
Nagorgani and Radha (2012)	2008	Introduction of regular fuzzy graphs
Akram (2011)	2011	Introduction of interval valued fuzzy graphs
Akram and Dudek (2011)	2011	Introduction of bipolar fuzzy graphs
Kittur (2012)	2012	Some properties of complete fuzzy graphs
Radha and Kumaravel (2014)	2014	Introduction of edge regular fuzzy graphs
Khansamy and Thangaraj (2015)	2015	Introduction of vertex-edge matrix of fuzzy graphs
This paper	–	Introduction of generalized fuzzy graphs Matrix representation of generalized fuzzy graphs Introduction of regular and complete generalized fuzzy graphs

Here, two types of relations are considered. In the following, generalized fuzzy graph of first kind is defined. Here, vertex membership values are considered first. Then, depending on vertex membership values, edge membership values are considered.

### **Definition 1**

Let *V* be a non-void set. Two functions are considered as follows: $\rho :V\rightarrow [0,1]$ and $\omega : V\times V\rightarrow [0,1]$. We suppose $A=\{(\rho (x),\rho (y))|\omega (x,y)> 0\}$. The triad $(V,\rho ,\omega )$ is defined to be generalized fuzzy graph of first kind (GFG1) if there exists a function $\phi :A\rightarrow (0,1]$ such that $\omega (x,y)=\phi ((\rho (x),\rho (y)))$ where $x,y\in V$. Here $\rho (x),x\in V$ is the membership value of the vertex *x* and $\omega (x,y),x,y\in V$ is the membership value of the edge (*x*, *y*).

### *Example 1*

**Fig. 1:**
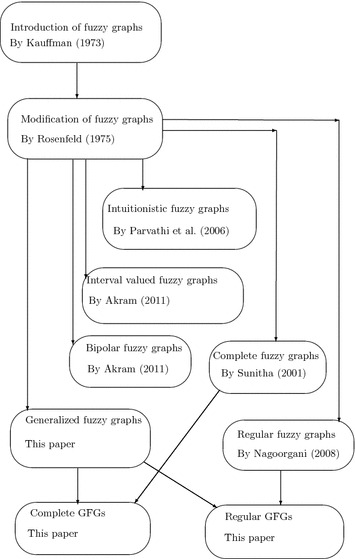
Flow chart of authors contribution

Let the vertex set be $V=\{x,y,z,t\}$ and edge set be {(*x*, *y*), (*x*, *z*), (*x*, *t*), (*y*, *t*)} and $\rho (x)=0.5$, $\rho (y)=0.9$, $\rho (z)=0.3$, $\rho (t)=0.8$. Let us consider $\phi (m,n)= m\vee n$. Here, *A* = {(0.5, 0.9), (0.5, 0.3), (0.5, 0.8), (0.9, 0.8)}. Then $\omega (x,y)=0.5$ $\vee 0.9=0.9$, $\omega (x,z)=0.5$, $\omega (x,t)=0.8$, $\omega (y,t)=0.9$. The corresponding generalized fuzzy graph is shown in Fig. 1.

Now, generalized fuzzy graphs of second kind is defined. Here, the membership values of edges are considered first. Then, depending on edge membership values, vertex membership values of vertices are assigned.

### **Definition 2**

Let *V* be a non-void set. Two functions are considered as follows: $\rho :V\rightarrow [0,1]$ and $\omega : V\times V\rightarrow [0,1]$ and let *B* be the range set of $\omega$. The triad $(V,\rho ,\omega )$ is defined to be generalized fuzzy graph of second kind (GFG2) if there exists a function $\psi :B\rightarrow (0,1]$ such that for every $x\in V$, $\rho (x)=\psi (\omega (e_x))$, where $e_x=(x,y)$ such that $y\in V$. Here, $\rho (x),x\in V$ is the membership values of the vertex *x* and $\omega (x,y)$ is the generalized membership value of the edge (*x*, *y*).

### *Note 1*

In GFG2, the co-domain set of $\psi$ excludes the number 0, as the membership values of vertices are always positive.

### *Example 2*

**Fig. 2:**
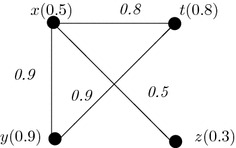
An example of generalized fuzzy graph of first kind (GFG1)

Let us consider a generalized fuzzy graph of second kind (GFG2) shown in Fig. 2. Here, $V=\{a,b,c,d,e\}$ be a non-void set and {((*a*, *b*), 0.4), ((*a*, *c*), 0.5), ((*a*, *e*), 0.6), ((*b*, *c*), 0.8), ((*b*, *e*), 0.3), ((*c*, *e*), 0.7), ((*d*, *e*), 0.7), ((*c*, *e*), 1)} is the fuzzy edge set. Also let, $\rho (x)=\frac{\sum _{y\in V}\omega (x,y)}{n}$ in which *n* is the whole number of existing edges adjacent to *x*. Now, $\rho (a)=\frac{0.4+0.5+0.6}{3}=0.5$. The vertex set is {*a*(0.5), *b*(0.5), *c*(0.75), *d*(0.85), *e*(0.575)}.

## Matrix representation of generalized fuzzy graphs

Any graph can be represented by a some well known matrices like adjacency matrices, incident matrices and many more (Khansamy and Thangaraj 2015). Among them, adjacent matrix and incident matrix are widely used. Fuzzy graphs can also be represented by these matrices. Here, two types of generalized fuzzy graphs are represented by two different types of matrices (Table 2).

**Table 2:** Matrix representation of GFG1

$\phi$	$v_1(\rho (v_1))$	$v_2(\rho (v_2))$	$\ldots$	$v_n(\rho (v_n))$
$v_1(\rho (v_1))$	$\rho (v_1)$	$\phi (\rho (v_1),\rho (v_2))$	$\ldots$	$\phi (\rho (v_1),\rho (v_n))$
$v_2(\rho (v_2))$	$\phi (\rho (v_2),\rho (v_1))$	$\rho (v_2)$	$\ldots$	$\phi (\rho (v_2),\rho (v_n))$
$\ldots$	$\ldots$	$\ldots$	$\ldots$	$\ldots$
$v_n(\rho (v_n))$	$\phi (\rho (v_n),\rho (v_1))$	$\phi (\rho (v_n),\rho (v_2))$	$\ldots$	$\rho (v_n)$

### Matrix representation of GFG1

This type of graph has one property that edge membership value depends on the membership values of adjacent vertices. Suppose $\xi =(V,\rho ,\omega )$ is a GFG1 where vertex set $V=\{v_1,v_2,\ldots ,v_n\}$. Now, $\phi :A\rightarrow (0,1]$ is taken such that $\omega (x,y)=\phi ((\rho (x),\rho (y)))$ where $x,y\in V$ and $A=\{(\rho (x),\rho (y))|\omega (x,y)> 0\}$. This graph can be represented by $(n+1)$ $\times$ $(n+1)$ matrix $M_{G_1}=[a(i,j)]$ as follows: in the first row and first column, vertices with membership values are provided. The (*i* + 1, *j* + 1)-th entry is the membership value of the edge $(x_i,x_j)$, $i,j=1,\ldots ,n$ if $i\ne j$. (*i*, *i*)-th entry is $\rho (x_i)$, where $i=1,2,\ldots ,n$. This membership value can be calculated easily using $\phi$ which is in (1, 1)-position of the matrix. The representation of the matrix is as follows:

$$\begin{aligned} a(1,1) & = \phi , \\ a(1,j + 1) & = x_{j} (\rho (x_{j} )),\quad {\text{where}}\;j = 1,2,3, \ldots ,n, \\ a(i + 1,1) & = x_{i} (\rho (x_{i} )),\quad \,{\text{where}}\;i = 1,2,3, \ldots ,n, \\ a(i + 1,j + 1) & = \omega (x_{i} ,x_{j} ),\quad \;{\text{where}}\;i,j = 1,2, \ldots ,n.\;{\text{and}}\;i \ne j \\ a(i + 1,i + 1) & = \rho (x_{i} ),\quad \quad \;\;{\text{where}}\;i = 1,2, \ldots ,n. \\ \end{aligned}$$

#### *Note 2*

1. The total information of GFG1 can be interpreted perfectly from the matrix representation.
2. The function $\phi$ is put in (1, 1)-position. Entries of the matrix are calculated from the function $\phi$ and vertex membership values which are put in 1st row and 1st column.
3. As matrices are considered for un-directed graphs, edges (*u*, *v*) and (*v*, *u*) are same and hence the matrices are symmetric.
4. Number of rows = number of columns = |*V*|.
5. If a row (column) has all its entries to be zero then that vertex is an isolated vertex.

#### *Example 3*

In this example, GFG1 of Fig. 2 is considered. Here, vertices of the graph are put along the rows and the columns. The corresponding matrix is shown in Table 3.

**Table 3:** An example of matrix representation of Fig. 2

$\phi (x,y)=\max \{x,y\}$	*x*(0.5)	*y*(0.9)	*z*(0.3)	*t*(0.8)
x(0.5)	0.5	0.9	0.5	0.8
y(0.9)	0.9	0.9	0	0.9
z(0.3)	0.5	0	0.3	0
t(0.8)	0.8	0.9	0	0.8

#### **Theorem 1**

*Let* $M_{G_1}$ *be the matrix representation of GFG1. Then* $D(x_k)=\sum _{j=1,j\ne k}^{n}a(k+1,j+1)$, $x_k\in V$ *or* $D(x_p)=\sum _{i=1,i\ne p}^{n}a(i+1,p+1)$, $x_p\in V$.

#### *Proof*

Here, $M_{G_1}$ is the matrix representation of GFG1. Then (i + 1, j + 1)-th entry is the membership value of the edge $(x_i,x_j)$, $i,j=1,\ldots ,n$ if $i\ne j$ i.e.,

$$a(i+1,j+1)=\omega (x_i,x_j),\quad {\text {where}}\quad i,j=1,2,\ldots ,n.\; {\hbox {and}}\quad i\ne j.$$

Now, degree of a vertex is the sum of the membership values of incident edges of the vertex. Thus, $D(x)=\sum _{y\in V}\omega (x,y)$. Again, the entries of a row or column are the membership values of corresponding edges except at diagonal entries. Hence, it is observed that $D(x_k)=\sum _{j=1,j\ne k}^{n}a(k+1,j+1)$, $x_k\in V$ or $D(x_p)=\sum _{i=1,i\ne p}^{n}a(i+1,p+1)$, $x_p\in V$. $\square$

### Matrix representation of GFG2

These types of graphs have one common property that edge membership value depends on adjacent vertex membership values. Suppose $\xi =(V,\rho ,\omega )$ is a GFG2, where vertex set is $V=\{v_1,v_2,\ldots ,v_n\}$ and $\{e_1,e_2,\ldots ,e_m\}$ is the edge set. Now, $\psi :B\rightarrow (0,1]$ is taken such that for every $x\in V$, $\rho (x)=\psi (\omega (x,y))$ where $y\in V$ and *B* is the range set of $\omega$. This graph can be represented by (*n* + 1) $\times$ (*m* + 2) matrix $M_{G_2}=[a(i,j)]$ as follows: here, edges with membership value are put in first row and vertices are put in first column. The (*i* + 1, *j* + 1)-th entry is either 1 or 0. If vertex $v_i$ is one of the end vertex of the edge $e_j$, then (*i* + 1, *j* + 1)-th entry is ‘1’ and ‘0’ otherwise. The (*m* + 2)-th column represents the membership values of the corresponding vertices. This value can be calculated by the function $\psi$, which is put in the (1, 1)-position. The matrix representation of the graph is determined as follows:

$$\begin{aligned} a(1,1) & = \psi \\ a(1,j + 1) & = e_{j} (\omega (e_{j} )),\quad {\text{where}}\;j = 1,2, \ldots ,m \\ a(i + 1,1) & = v_{i} ,\quad {\text{where}}\;i = 1,2, \ldots ,n \\ a(i + 1,j + 1) & = \left\{ {\begin{array}{*{20}l} {1,} \hfill &\quad {{\text{if}}\;{\text{j - th}}\;{\text{edge}}\;e_{j} \;{\text{is}}\;{\text{incident}}\;{\text{on}}\;{\text{i - th}}\;{\text{vertex}}\;v_{i} ,{\text{For}}\;i,j = 1,2, \ldots ,n} \hfill \\ {0,} \hfill &\quad {{\text{otherwise,}}\;{\text{For}}\;i,j = 1,2, \ldots ,n.} \hfill \\ \end{array} } \right. \\ a(i + 1,m + 2) & = \rho (v_{i} ),\quad {\text{where}}\;i = 1,2, \ldots ,n \\ \end{aligned}$$

#### *Note 3*

1. The total information of GFG2 can be interpreted perfectly from the matrix representation.
2. The function $\psi$ is put in (1, 1)-position. Entities of last column of the matrix are calculated from the function $\psi$ and edge membership values which are put in corresponding positions of 1st row.
3. If a row (column) has all its entries to be zero, then that vertex is an isolated vertex.

#### *Example 4*

**Fig. 3:**
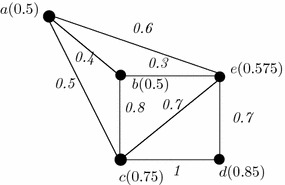
An example of generalized fuzzy graph of second kind (GFG2)

Let us consider the GFG2 of Fig. 3. Here, the edges are assigned as $(a,e)\rightarrow e_1$, $(a,b)\rightarrow e_2$, $(a,c)\rightarrow e_3$, $(b,c)\rightarrow e_4$, $(c,d)\rightarrow e_5$, $(d,e)\rightarrow e_6$, $(e,b)\rightarrow e_7$, $(c,e)\rightarrow e_8$. In this graph, $\psi (x_1,x_2,\ldots ,x_k)=\frac{\sum _{i=1}^kx_i}{k}$ is assumed. The calculation is shown in Table 4.

**Table 4:** Matrix representation of GFG2

$\psi$	$e_1(\omega (e_1))$	$e_2(\omega (e_2))$	$\ldots$	$e_m(\omega (e_m))$	$\rho (v)$
$v_1$	0	1	…	0	$\psi (\omega (e_1))$
$v_2$	1	1	…	1	$\psi (\omega (e_1),\omega (e_2),\ldots ,\omega (e_1))$
…	…	…	…	…	…
$v_n$	1	0	…	1	$\psi (\omega (e_1),\omega (e_m))$

First row of the matrix indicates the existence of vertices among the edges. Here, the vertex ‘a’ is incident to the edges $e_1,e_2,e_3$. Hence, ‘1’ is put in the corresponding columns and ‘0’ in the remaining columns. The membership value of the vertices will be calculated from the function $\psi (x_1,x_2,\ldots ,x_k)=\frac{\sum _{i=1}^kx_i}{k}$, where $x_i$, $i=1,2,\ldots ,k$ are the edge membership values.

#### **Theorem 2**

*Let* $M_{G_2}$ *be the* $(n+1)$ *by* $(m+2)$ *matrix of GFG2,* $\xi$ *. Also let,* $i+1$ *-th row has ‘1’ as entries in* $a(i+1,m_1),a(i+1,m_2),\ldots , a(i+1,m_p)$ *positions where* $i=1,2,\ldots , n$ *. Then,* $a(i+1,m+2)=\phi (e_{m_1},e_{m_2},\ldots ,e_{m_p})$.

#### *Proof*

Let $\xi =(V,\rho ,\omega )$ be a GFG2 with $\psi :B\rightarrow (0,1]$ such that for every $x\in V$, $\rho (x)=\psi (\omega (x,y))$ where $y\in V$. Here, $M_{G_2}$ is a $(n+1)$ by $(m+2)$ matrix of $\xi$. Thus,

$$a(i+1,j+1)= \left\{ \begin{array}{ll} 1, &{}\text { if } j\text {-th edge } e_j {\text { is incident on }} i{\text {-th vertex }} v_i\\ 0, &{}{\text { otherwise}}. \end{array} \right.$$

Now, each row has ‘0’ or ‘1’ as entries. Without loss of generality, $(i+1)$-th row has ‘1’ as entry in $a(i+1,m_1),a(i+1,m_2),\ldots , a(i+1,m_p)$ positions. Now, ‘1’ as entry, means the vertex $x_i$ is incident with the corresponding edge of column. Thus, the vertex $x_i$ is incident with the edges $e_{m_1},e_{m_2},\ldots ,e_{m_p}$. Thus, $\rho (x_i)=\psi (e_{m_1},e_{m_2},\ldots ,e_{m_p}).$ $\square$

## Complete generalized fuzzy graphs

In general, if the membership value of an edge is greater than half of its maximum of membership values of its end vertices, the edge is called to be an effective edge. Suppose, someone informs another person about any news. If the transfer of knowledge is greater than to a certain amount (may be assumed half of the source knowledge), then the second person can be informed effectively. Thus, transfer of knowledge (which indicates the membership value of an edge) helps a person (a vertex with lower membership value) to be informed from a source (a vertex with greater membership value). Definition of an effective edge is given below.

### **Definition 3**

Let $\xi =(V,\rho ,\omega )$ be a GFG1 (or GFG2). An edge (*x*, *y*) is defined to be effective edge if $\omega (x,y)\ge \frac{1}{2}\max \{\rho (x),\rho (y)\}$. A generalised fuzzy graph GFG1 (or GFG2) is defined to be effective, if for all $x,y\in V$, $\omega (x,y)\ge \frac{1}{2}\max \{\rho (x),\rho (y)\}$.

### *Note 4*

It is obvious that for GFG1, $\omega (x,y)=\phi (x,y)$. Now, if an edge of a generalized fuzzy graph is effective, then others edge may not be effective. The following is the analytic description of this statement. Let $\xi$ be a GFG1 and it has vertex set $\{a(0.8),b(0.2),c(0.3)\}$ and edge set $\{(a,b),(b,c)\}$ and $\phi (x,y)=\min \{x,y\}$. Then, it can be found $\phi (a,b)=0.2,\phi (b,c)=0.2$. The edge (*b*, *c*) *is effective as* $\phi (b,c)=0.2>\frac{1}{2}max\{b,c\}=1.5$. Thus, the edge (*a*, *b*) is not effective.

A simple graph *G* is defined to be complete if every vertex in *G* is connected with every other vertex, i.e., if *G* contains only one edge between each pair of distinct vertices. Now, we have to check the completeness of generalized fuzzy graphs. Now, it is easy to consider that an edge is called to be non-effective if it is not effective. In that case, the edge may be ignored for some representation. Thus to consider that a generalized fuzzy graph is complete, effectiveness of edges are important. The definition of complete generalized fuzzy graph is defined below.

### **Definition 4**

Let $\xi =(V,\rho ,\omega )$ be a GFG1 (or GFG2). The graph $\xi$ is defined to be complete if all pairs of vertices are connected by effective edges. Otherwise, the graph is defined to be incomplete generalized fuzzy graph.

### *Example 5*

**Fig. 4:**
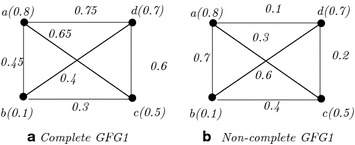
Complete and non-complete generalized fuzzy graphs

Let $\xi$ be a GFG1 with vertex set $\{a(0.8), b(0.1),c(0.5),d(0.7)\}$ and edge set $\{(a,b),(a,c),(a,d),(b,c),(b,d),(c,d)\}$ and $\phi (x,y)=\frac{x+y}{2}$. Then, the membership values of the edges (*a*, *b*), (*a*, *c*), (*a*, *d*), (*b*, *c*), (*b*, *d*), (*c*, *d*) are 0.45, 0.65, 0.75, 0.3, 0.4, 0.6, respectively (see Fig. 4a). Then, $\xi$ is complete as all the edges are effective.

### *Note 5*

If we change the function $\phi$ of Fig. 4 and redefine the function $\phi (x,y)=|x-y|$, then the graph is not complete as some of its edges are non-effective (see Fig. 4b).

### **Theorem 3**

Let $\xi =(V,\rho ,\omega )$ be a complete GFG1 or GFG2. Then $D(x)\ge \frac{|V|-1}{2}\rho (a)$ for all $x\in V$.

### *Proof*

Here, $\xi =(V,\rho ,\omega )$ be a complete GFG1 or GFG2. From the definition of complete GFG1 or GFG2, every vertex is connected with all remaining vertices, i.e. $(|V|-1)$ vertices by effective edges. Now, effective edges have a property that $2\omega (x,y)\ge \max \{\rho (x),\rho (y)\}$. Hence, $D(x)=$ sum of membership values of all adjacent edges of *x* which is obviously greater than $\frac{|V|-1}{2}\rho (a)$. Hence, it proves Theorem 3. $\square$

## Regularity of generalized fuzzy graphs

**Fig. 5:**
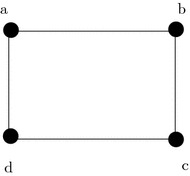
Crisp regular graph of degree 2

A graph, with all vertices are of equal degree, is defined to be a regular graph. If each vertex is of degree *r*, then the graph is defined to be a regular graph with degree *r*. For example, in Fig. 5, a regular graph with degree 2 is shown.

The degree of a vertex in generalized fuzzy graph is defined here. Let $\xi =(V,\rho ,\omega )$ be a generalized fuzzy graph (GFG1 or GFG2). Now, the degree of a vertex *x* is denoted as $D(x)=\sum _{y\in V}\omega (x,y)$. The regularity of a generalized fuzzy graph means that the degrees of each vertex is same. But, for large networks, it is difficult to confirm that all vertices have same degree. If major number of vertices have the same degree or almost same degree, then the graph is defined to be regular. The definition of the regular generalized fuzzy graphs is given below (Table 5).

**Table 5:** An example of matrix of Fig. 3

$\psi$	$e_1(0.6)$	$e_2(0.4)$	$e_3(0.5)$	$e_4(0.8)$	$e_5(1)$	$e_6(0.7)$	$e_7(0.3)$	$e_8(0.7)$	$\rho (x)$
a	1	1	1	0	0	0	0	0	0.5
b	0	1	0	1	0	0	1	0	0.5
c	0	0	1	1	1	0	0	1	0.75
d	0	0	0	0	1	1	0	0	0.85
e	1	0	0	0	0	1	1	1	0.575

### **Definition 5**

Let $\xi =(V,\rho ,\omega )$ be a GFG1 (or GFG2). Now, degree of a vertex *x* in $\xi$ is denoted as *D*(*x*) and is defined as $D(x)=\sum _{y\in V}\omega (x,y))$. Also, let $k=\frac{\sum _{x\in V}{D(x)}}{n}$, where *n* is the whole number of existing elements of *V*. Now $\xi$ is defined to be regular if for every $x\in V$, $|D(x)-k|\le \epsilon$, where $\epsilon$ is very small number and $0\le \epsilon \le 1$.

In this study, $\epsilon$ is assumed as the upper bound of $|D(x)-k|$. This value may vary for different networks and it is decided by decision makers. This graph can be called $\epsilon$-regular generalized fuzzy graphs.

### *Note 6*

If $\epsilon =0$, then $D(x)=k$ for all $x\in V$. Hence, it is called regular GFGs i.e. 0-regular GFGs are regular GFGs.

### *Example 6*

**Fig. 6:**
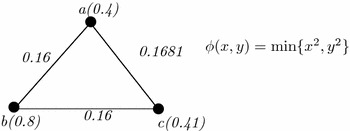
A non-regular GFG1

Let, $\xi$ be a GFG1 vertex set $\{a(0.4),b(0.8),c(0.41)\}$. Let $\phi (x,y)=\min \{x^2,y^2\}$. Thus, the membership values of (*a*, *b*), (*b*, *c*), (*a*, *c*) are 0.16, 0.168, 0.16 respectively (see Fig. 6). Now, the degree of the vertices are *D*(*a*) = 0.32, *D*(*b*) = 0.328, *D*(*c*) = 0.328. $k=\frac{0.32+0.328+0.328}{3}=0.325$. Let us take $\epsilon =0.00325$. Now, $|D(a)-k|=0.005>0.00325$, $|D(b)-k|=0.003<0.00325$ and $|D(c)-k|=0.003<0.00325$. Thus, the graph is not regular as degree of ‘*a*’ does not satisfy the condition. Although other vertices satisfy the condition. If $\epsilon$ is decreased, then the graph may be regular.

### **Theorem 4**

*Let* $\xi$ *be* $\epsilon$ *-regular GFG1. Also, let the corresponding crisp graph be a cycle. Suppose, the length of the crisp cycle is odd. Then,* $\sup A-\inf A\le 2\epsilon$ *, where* $A=\{\phi (x,y): x,y\in V\}$.

### *Proof*

**Fig. 7:**
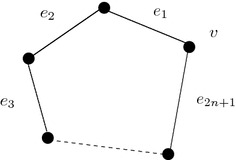
A cycle of odd length

Let $\xi =(V,\rho , \omega )$ be a $\epsilon$-regular fuzzy graphs. Thus, $|D(x)-k|\le \epsilon$ for all $x\in V$, where $k=\frac{\sum _{x\in V}{D(x)}}{n}$ in which *n* is the whole number of existing elements of *V*. Here, it is considered as $A=\{\phi (x,y): x,y\in V\}$. Now, let $e_1,e_2,\ldots ,e_{2n+1}$ be the edges of the graph (see Fig. 7). As, the supremum and infimum are to be found out for this proof, the maximum/minimum value of the range set of $\phi$ are considered without loss of generality. Let $\phi (e_1)=k_1$, then $\phi (e_2)=k-k_1\pm \epsilon$. $\phi (e_3)=k-(k-k_1\pm \epsilon )$ $=k_1\pm \epsilon$, thus, $\phi (e_{2n+1})=k_1\pm \epsilon$. If *v* is the connecting vertex of $e_1$ and $e_{2n+1}$, then $D(v)=\phi (e_1)+\phi (e_{2n+1})$ $=k\pm \epsilon$. Thus, it can be found as $k_1+k_1\pm \epsilon =k\pm \epsilon$ which gives $k_1=\frac{k}{2}$ or $\frac{k}{2}+\epsilon$ or $\frac{k}{2}-\epsilon$. Thus, $\sup A-\inf A\le 2\epsilon$. $\square$

### *Note 7*

The converse of the Theorem 4 is not necessarily true. If the condition of edge of any cycle is true, the cycle need not be regular.

### **Theorem 5**

*Let* $\xi$ *be* $\epsilon$ *-regular GFG1. Also, let the corresponding crisp graph be a cycle. Suppose the length of the cycle is even. Then,* $\sup A-\inf A\le 2\epsilon$ *where* $A=\{\phi (x,y): x,y\in V\}$ *or* $\sup B-\inf B\le 2\epsilon$ *, where* $B=\{\phi (e)$ *: for all alternating edges e of the cycle* $\xi \}$.

### *Proof*

Let $\xi =(V,\rho , \omega )$ be a $\epsilon$-regular fuzzy graphs. Thus, $|D(x)-k|\le \epsilon$ for all $x\in V$ and $A=\{\phi (x,y): x,y\in V\}$. Now, let $e_1,e_2,\ldots ,e_{2n}$ be the edges of the graph (see Fig. 7). As, the supremum and infimum are to be found out for this proof, the maximum/minimum value of the range set of $\phi$ are considered without loss of generality. Let $\phi (e_1)=k_1$ then, $\phi (e_2)=k-k_1\pm \epsilon$, $\phi (e_3)=k-(k-k_1\pm \epsilon$ $=k_1\pm \epsilon$, thus, it is found $\phi (e_{2n})=k-k_1\pm \epsilon$. If *u* is the connecting vertex of $e_1$ and $e_{2n}$, $D(u)=\phi (e_1)+\phi (e_{2n})$ $=k\pm \epsilon$. Thus, proceeding the concept of Theorem 4, it is found $\sup A-\inf A\le 2\epsilon$, where $A=\{\phi (x,y): x,y\in V\}$. Besides, for odd edges maximum/minimum value of $\phi (e)=k_1\pm \epsilon$ and for even edges $\phi (e)=k-k_1\pm \epsilon$. Hence, $\sup B-\inf B\le 2\epsilon$, where $B=\{\phi (e): \text { for all alternating edges e of the cycle } \xi \}$. $\square$

### *Note 8*

The size of generalized fuzzy graphs (GFG1 or GFG2) is the sum of membership values of the edges. Again, the twice of the size of a fuzzy graph is equal to the aggregate of the degrees of vertices. Now, for $\epsilon$-regular GFG1 or GFG2 with n vertices, size of the graph has upper bound as $\frac{n(k+ \epsilon )}{2}$ and lower bound as $\frac{n(k- \epsilon )}{2}$. Thus, if size of a GFG1 or GFG2, $\xi$ is denoted by $S(\xi )$, then

$$\frac{n(k-\epsilon )}{2}\le S(\xi )\le \frac{n(k+\epsilon )}{2}.$$

**Fig. 8:**
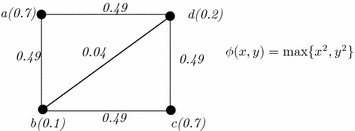
An effective regular GFG1

Effective edges have significant values in every systems. Thus, only these edges are counted for degree of a vertex and the definition of effective degree is given below (Fig. 8).

### **Definition 6**

Let $\xi =(V,\rho ,\omega )$ be a GFG1 (or GFG2) and let *E* be the set of all effective edges of $\xi$. Now, effective degree of a vertex *x* in $\xi$ is denoted as $\mathcal {D}(x)$ and is defined as $\mathcal {D}(x)=\sum _{(x,y)\in E,y\in V}\phi (x,y)$.

If all the vertices of a GFG1 or GFG2 are of same effective degree or almost same effective degree, the graph is defined to be effective regular graph. The definition is given in the following:

### **Definition 7**

Let $\xi =(V,\rho ,\omega )$ be a GFG1 (or GFG2) and let *E* be the set of all effective edges of $\xi$ and $k=\frac{\sum _{(x,y)\in E}{\mathcal {D}(x)}}{n}$ in which *n* is the whole number of elements of *V*. Now $\xi$ is defined to be $\epsilon$-effective regular if for all $x\in V$, $|\mathcal {D}(x)-k|\le \epsilon$.

### *Example 7*

Let us consider a GFG1, $\xi$ with vertex set $\{a(0.7),b(0.1),c(0.7),d(0.2)\}$ and $\phi (x,y)=\max \{x^2,y^2\}$. Thus, the edge membership values of (*a*, *b*), (*b*, *c*), (*c*, *d*), (*d*, *a*), (*b*, *d*) are 0.49, 0.49, 0.49, 0.49, 0.04 respectively. Now by calculation, it can be observed that (*b*, *d*) is not effective. Thus, effective degrees of the vertices are same and equal to 0.98. Thus, the graph is $\epsilon$-effective regular GFG1.

In the definition of $\epsilon$-effective regular generalized graphs, $\epsilon$ is assumed as positive real number. But in the following definition, *p* is assumed a positive integer.

### **Definition 8**

Let $\xi =(V,\rho ,\omega )$ be a GFG1 or GFG2. $\xi$ is defined to be *p*-regular effective generalized fuzzy graph if every vertex of $\xi$ is incident to exactly *p* number of effective edges.

### *Example 8*

**Fig. 9:**
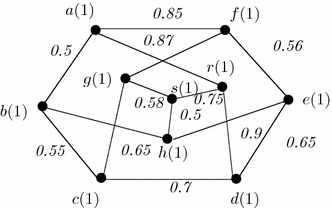
A 3-regular effective generalized fuzzy graph

A generalized graph is shown in Fig. 9. All vertices has 3 adjacent vertices. Thus, this graph can be called as 3-regular effective generalized fuzzy graphs.

### **Theorem 6**

*Let* $\xi =(V,\rho ,\omega )$ *be a p-regular effective GFG1 or GFG2, then* $D(x)\ge \frac{p-1}{2}\rho (x)$.

### *Proof*

Proof of this theorem is obvious, keeping the reference of the proof of Theorem 3. $\square$

### **Theorem 7**

Every complete generalized fuzzy graph $\xi =(V,\rho ,\omega )$ (GFG1 or GFG2) is $(|V|-1)$-regular effective generalized fuzzy graph.

### *Proof*

Let $\xi =(V,\rho ,\omega )$ be a complete GFG1 or GFG2. Now, every vertex of a complete GFG1 or GFG2 is adjacent to remaining $(|V|-1)$ vertices. By the definition of *p*-effective regular fuzzy graphs, it is easy to verify that $\xi$ is $(|V|-1)$-regular effective generalized fuzzy graph. $\square$

## Insights of this study

- Fuzzy graphs are generalized with removal of the edge restriction. Thus any kinds of networks can be represented by GFGs.
- Vertex and edge relation of GFGs are established.
- Matrix representation of GFGs are given. This is the easier way to represent any GFGs.
- Two major properties of GFGs, completeness and regularity, are provided. Some important results are proved.

## Conclusions

This study described some major properties of generalized fuzzy graphs. In the literature, adjacent matrices and incident matrices are available. Here, GFG1 was represented by matrices, which was similar to adjacent matrices of fuzzy graphs. But, the difference is that each element is determined from the function $\phi$. Again, GFG2 was represented by matrices similar to incident matrices. These representations are helpful to understand the generalized fuzzy graphs in easier way. This study also introduced two properties namely, completeness and regularity of GFG1 or GFG2. Effective regular and *p*-effective regular GFG1 or GFG2 were described. Some results regarding the definitions were established. In near future, eigenvalues of matrices and their properties will be established (Gholmy and Hawary 2016). This study will develop the theory of fuzzy graphs along with some important algorithms and networking problems (Oghlan et al. 2016).
